# Evaluation of a social franchising and telemedicine programme and the care provided for childhood diarrhoea and pneumonia, Bihar, India

**DOI:** 10.2471/BLT.16.179556

**Published:** 2017-03-24

**Authors:** Manoj Mohanan, Soledad Giardili, Veena Das, Tracy L Rabin, Sunil S Raj, Jeremy I Schwartz, Aparna Seth, Jeremy D Goldhaber-Fiebert, Grant Miller, Marcos Vera-Hernández

**Affiliations:** a Sanford School of Public Policy, Duke University, 302 Towerview Drive, 128 Rubenstein Hall, Durham, North Carolina, NC 27708, United States of America (USA).; b Department of Economics, Queen Mary University of London, London, England.; c Department of Anthropology, Johns Hopkins University, Baltimore, USA.; d Department of Internal Medicine, Yale University School of Medicine, New Haven, USA.; e Indian Institute of Public Health, New Delhi, India.; f Sambodhi Research and Communications Pvt. Ltd., New Delhi, India.; g Center for Health Policy and Center for Primary Care and Outcomes Research, Stanford University School of Medicine, Stanford, USA.; h Department of Economics, University College London, London, England.

## Abstract

**Objective:**

To evaluate the impact on the quality of the care provided for childhood diarrhoea and pneumonia in Bihar, India, of a large-scale, social franchising and telemedicine programme – the World Health Partners’ Sky Program.

**Methods:**

We investigated changes associated with the programme in the knowledge and performance of health-care providers by carrying out 810 assessments in a representative sample of providers in areas where the programme was and was not implemented. Providers were assessed using hypothetical patient vignettes and the standardized patient method both before and after programme implementation, in 2011 and 2014, respectively. Differences in providers’ performance between implementation and nonimplementation areas were assessed using multivariate difference-in-difference linear regression models.

**Findings:**

The programme did not significantly improve health-care providers’ knowledge or performance with regard to childhood diarrhoea or pneumonia in Bihar. There was a persistent large gap between knowledge of appropriate care and the care actually delivered.

**Conclusion:**

Social franchising has received attention globally as a model for delivering high-quality care in rural areas in the developing world but supporting data are scarce. Our findings emphasize the need for sound empirical evidence before social franchising programmes are scaled up.

## Résumé

**Objectif:**

Évaluer l'impact d'un programme à large échelle de franchises sociales et de télémédecine -le programme « Sky » de WHP (World Health Partners)- sur la qualité de la prise en charge de la diarrhée et de la pneumonie infantiles dans le Bihar, en Inde.

**Méthodes:**

Nous avons étudié les changements associés à ce programme en termes de connaissances et de performances des prestataires de soins de santé, en réalisant 810 évaluations sur un échantillon représentatif de prestataires de soins, dans des régions couvertes et non-couvertes par le programme. Les prestataires ont été évalués en utilisant des scénarios de patients hypothétiques et la méthode du patient standardisé, avant et après la mise en œuvre du programme, en 2011 et 2014 respectivement. Les différences constatées dans les performances des prestataires entre les régions couvertes et non-couvertes par le programme ont été évaluées avec des modèles multivariés de régression linéaire de l'écart des différences.

**Résultats:**

Le programme n'a pas sensiblement amélioré les connaissances ou les performances des prestataires de soins en lien avec les deux maladies infantiles étudiées, dans le Bihar. En revanche, notre étude montre qu'un gros écart persiste entre la connaissance des soins appropriés et les soins effectivement dispensés.

**Conclusion:**

Les franchises sociales retiennent actuellement l'attention du monde entier en tant que solution envisageable pour obtenir des soins de grande qualité dans les régions rurales des pays en développement, mais les données probantes sont encore rares. Nos résultats soulignent la nécessité d'obtenir des données empiriques solides avant d'intensifier le déploiement de programmes de franchises sociales.

## Resumen

**Objetivo:**

Evaluar el impacto en la calidad de la atención proporcionada para la diarrea y la neumonía infantiles en Bihar, India, de un programa de telemedicina y franquicia social a gran escala, el programa Sky de la ONG World Health Partners.

**Métodos:**

Se investigaron los cambios relacionados con el programa en el conocimiento y el rendimiento de los proveedores de atención de salud realizando 810 evaluaciones en una muestra representativa de proveedores en zonas en las que el programa se implementó y en zonas en las que no. Los proveedores fueron evaluados a través de viñetas de pacientes hipotéticos y el método del paciente estandarizado antes y después de la implementación del programa, en 2011 y 2014, respectivamente. Se evaluaron las diferencias en cuanto al rendimiento de los proveedores entre las zonas de implementación y las de no implementación utilizando unos modelos de regresión lineal multivariables de diferencias en diferencias.

**Resultados:**

El programa no mejoró de forma significativa el conocimiento ni el rendimiento de los proveedores de atención de salud en cuanto a la diarrea o la neumonía infantil en Bihar. El resultado fue una gran y constante diferencia entre el conocimiento de la atención adecuada y la que se proporcionaba.

**Conclusión:**

La franquicia social ha recibido atención a escala mundial como un modelo para proporcionar una atención de alta calidad en zonas rurales del mundo en desarrollo, aunque los datos que lo fundamentan son escasos. Los resultados destacan la necesidad de una prueba empírica adecuada antes de ampliar los programas de franquicia social.

## ملخص

**الهدف ** تقييم التأثير على نوعية الرعاية المقدمة لحالات الإصابة بالإسهال والالتهاب الرئوي بين الأطفال في بيهار بالهند، من خلال برنامج واسع النطاق لشبكات خدمات الرعاية الاجتماعية والعلاج الطبي عن بُعد، وهو المتمثل في برنامج سكاي التابع لمنظمة "وورلد هيلث بارتنرز".

**الطريقة:**

قمنا بالتحقيق في التغييرات المرتبطة بالبرنامج من حيث معرفة وأداء مقدمي الرعاية الصحية من خلال تنفيذ 810 تقييمات في عينة تمثيلية لمقدمي الرعاية في المناطق التي تم تنفيذ البرنامج فيها والمناطق الأخرى التي لم يتم التنفيذ فيها. وتم تقييم مقدمي الرعاية باستخدام توصيفات افتراضية للمرضى والطريقة المعيارية للتعامل مع المرضى قبل تنفيذ البرنامج وبعده، وذلك في عامي 2011 و2014 على التوالي. وتم تقييم الاختلافات في أداء مقدمي الرعاية بين المناطق التي تم فيها التنفيذ والمناطق التي لم يمتد إليها التنفيذ، وذلك باستخدام نماذج الانحدار الخطي متعددة المتغيرات بأسلوب قياس الاختلاف المتباين.

**النتائج:**

لم يحقق البرنامج تحسنًا كبيرًا في معرفة مقدمي الرعاية الصحية ولا أدائهم فيما يتعلق بحالات الإصابة بالإسهال أو الالتهاب الرئوي بين الأطفال في بيهار. وكانت هناك فجوة كبيرة ومستمرة بين معرفة الرعاية المناسبة والرعاية المقدمة بالفعل.

**الاستنتاج:**

حظيت شبكات خدمات الرعاية الاجتماعية بالاهتمام على المستوى العالمي كنموذج لتقديم الرعاية عالية الجودة في المناطق الريفية في العالم النامي ولكن البيانات الداعمة كانت شحيحة. وتؤكد نتائجنا على الحاجة إلى أدلة تجريبية سليمة قبل توسيع نطاق شبكات خدمات الرعاية الاجتماعية. ****

## 摘要

**目的:**

旨在评估大型社会连锁远程医疗项目——世界卫生伙伴天空计划对印度比哈尔邦地区儿童腹泻与肺炎护理质量的影响。

**方法:**

通过对该项目实行及未实行地区医疗护理提供者的代表性样本开展 810 次评估，我们调查了提供者在知识与表现方面与该项目相关的变化。 分别在 2011 年与 2014 年，即该项目实行前后，通过采用假定患者情境与标准化患者方法对提供者进行评估。 通过采用多元线性回归模型双重差分析法对实行与未实行地区提供者表现的差异进行评估。

**结果:**

该项目未能显著提升比哈尔邦地区儿童腹泻或肺炎卫生护理提供者的知识或表现。 得当的护理知识与实际提供的护理之间仍存在巨大差距。

**结论:**

社会连锁项目，作为一种为发展中国家乡村地区提供高质量护理的模型，已获得了全球的关注，但缺乏支持性数据。 我们的结果突出了扩大社会连锁项目之前对完备的经验性证据的需求。

## Резюме

**Цель:**

Оценить влияние крупномасштабной программы социального франчайзинга и телемедицины — программы Sky международной некоммерческой организации World Health Partners (Партнеры в области здравоохранения) — на качество медицинского обслуживания при детской диарее и пневмонии в штате Бихар, Индия.

**Методы:**

Авторы исследовали изменения, связанные с программой, в уровне знаний и показателях эффективности работы медицинских работников, проведя 810 оценок в репрезентативной выборке медицинских работников в тех областях, где программа была и где не была реализована. Оценка медицинских работников проводилась с помощью виньеток (т. е. сценариев) с гипотетическими пациентами и метода, использующего стандартизованных пациентов, до и после реализации программы в 2011 и 2014 годах соответственно. Различия в показателях эффективности работы медицинских работников между областями, где программа была и где не была реализована, оценивались с использованием многофакторных моделей линейной регрессии методом «разность разностей».

**Результаты:**

Программа не оказала значительного влияния на уровень знаний медицинских работников или эффективность их работы в отношении детской диареи или пневмонии в штате Бихар. Наблюдался постоянный большой разрыв между знанием о надлежащем уходе и фактически оказанной медицинской помощью.

**Вывод:**

Социальный франчайзинг привлек общемировое внимание как модель для оказания высококачественной помощи в сельских районах в развивающихся странах, но при этом подкрепляющих данных недостаточно. Сделанные выводы подчеркивают необходимость получения надежных эмпирических данных до того, как будет расширен масштаб программ социального франчайзинга.

## Introduction

Diarrhoea and pneumonia are leading causes of childhood morbidity and mortality despite being targeted by global investment in health for decades. In the last 5 years, these diseases were responsible for almost 25% of deaths among children aged 1 to 4 years worldwide.^1^ In India, nearly 500 000 children younger than 5 years died from diarrhoea or respiratory infections in 2013.^2^ These poor health outcomes were partially due to the low quality of care in both public and private health sectors, including absenteeism, poor knowledge and the know–do gap, i.e. the gap between knowledge of appropriate care and the care actually delivered.^3^^–^^12^

Efforts to improve the quality of primary care in developing countries have focused on a variety of strategies, ranging from training and performance incentives to organizational innovations in the private sector and the use of new technologies, such as telemedicine.^13^ One prominent approach is social franchising, which is similar to commercial franchising. The aim is to improve a socially desirable outcome, such as health, while generating sufficient revenue to be self-sustaining. In social franchising, a franchisor offers a standardized, branded set of products or services through franchisees who pay a subscription fee to join the franchisor’s network. Franchisees, who are typically existing local providers, in turn, receive training and follow service delivery protocols established by the franchisor. They also benefit from marketing, branding, supply chain management and diagnostic services organized by the franchisor. Many franchisors use new information technologies to improve the efficiency and coordination of their network of franchisees. However, despite the rapid growth of social franchising in developing countries, there is little evidence that it affects the quality of care provided on a large scale,^14^^–^^16^ except for a recent programme in Myanmar where the introduction of social franchising was accompanied by a substantial increase in the number of health-care workers.^17^^,^^18^

In this study, we investigated the effectiveness of the World Health Partners’ Sky Program in improving the knowledge and performance of health-care providers in Bihar, India. In addition to its effect on participating health-care providers, it was hoped that the programme would also improve the performance of other local providers through spillover effects and by encouraging competition. Understanding of the programme’s effect on the quality of care could help to explain why a programme in rural Bihar costing over 23 million United States dollars (US$) failed to increase the appropriate treatment of childhood diarrhoea or pneumonia.^19^

The programme was originally developed as a hub-and-spoke model: SkyHealth providers with telemedicine facilities at the hub would link to peripheral rural health providers with smaller, more basic facilities (called SkyCare providers). SkyCare providers typically offered basic primary care and symptom-based treatment and could refer patients to SkyHealth providers.^20^ SkyHealth providers had access to telemedicine technology that was able to connect physicians at the World Health Partners’ central medical facility in Delhi to patients in rural areas via an audiovisual interface. Using this technology, physicians were able to examine patients by auscultation, assess their blood pressure and pulse and obtain electrocardiogram results if needed. In addition, SkyCare providers could offer mobile phone consultations with these physicians or refer patients to SkyHealth providers.

Although planned as a hub-and-spoke model in which SkyHealth providers would be responsible for the empanelment of SkyCare providers, the programme was instead implemented as a two-tier model without referral networks in which the two types of provider were recruited directly by World Health Partners. Existing informal health-care providers or pharmacists in rural areas were approached by World Health Partners’ field representatives, given information about the programme and offered the opportunity to join the network by paying a franchisee fee. In early 2014, the franchisee fee for a SkyHealth centre was US$ 500 in addition to an investment of approximately US$ 1000 to set up the telemedicine centre.^21^ SkyCare providers paid a franchisee fee of US$ 17 plus a small fee of US$ 0.17 for each mobile phone consultation. The training offered reflected the heterogeneity of providers in the network: SkyHealth providers received 6 days of training on the diagnosis and treatment of infectious diseases, whereas training lasted 3 days for SkyCare providers.^21^ Both types of provider received some training on protocols for basic service delivery, marketing services, diagnostic services and ensuring a predictable supply of adequate-quality drugs. SkyHealth providers were also trained in operating computers for telemedicine services.

## Methods

We assessed the knowledge and performance of health-care providers in 80 study clusters across 11 districts in Bihar at baseline between June and September 2011 and again at follow-up between June and September 2014. These 80 clusters were randomly selected, using Stata version 13.1 (StataCorp. LP, College Station, United States of America) from the 360 study clusters included in the Bihar Evaluation of Social Franchising and Telemedicine project before the Sky Program was implemented.^22^ Clusters comprised catchment areas surrounding a central village that met eligibility criteria for the possible establishment of a SkyHealth telemedicine centre – criteria primarily included the availability of a broadband connection, health-care infrastructure and potential investors in the franchisee network.^20^ To evaluate the effect of the Sky Program on the quality of care provided to households, we interviewed a sample of 64 households in each study cluster and selected the five most frequently visited providers for our baseline sample (i.e. we implicitly randomly selected providers on the basis of their market share).^19^ This process was repeated at follow-up but we also added providers from the Skycare programme network if they were not among the five most frequently visited providers at follow-up. In total, we carried out 810 assessments: 395 at baseline and 415 at follow-up. We collected information on the health-care provider’s age, educational attainment, socioeconomic characteristics and health-care practice and background, including medical training and experience, types of illness treated and familiarity with technology. At recruitment, providers consented to taking part in vignette interviews and to being visited unannounced by standardized patients within the following 2 months. Our primary outcomes were: (i) providers’ knowledge of the appropriate treatment for childhood diarrhoea and pneumonia as assessed using vignettes; and (ii) providers’ performance in dealing with cases of these diseases as assessed using standardized patients. Our protocols and instruments are available from the corresponding author on request. This study, which formed part of the Bihar Evaluation of Social Franchising and Telemedicine project protocol, was approved by Duke University in the United States of America (approval no. 29755) and the Health Ministry Steering Committee in India (No.12/2008/30-HMSC/4).

### Vignettes

Two interviewers presented hypothetical patient vignettes to health-care providers. One interviewer recorded the diagnostic questions asked by providers in response to the vignettes and the other one read scripted responses to these questions.^23^^–^^25^ Vignettes were devised to represent cases of childhood diarrhoea and pneumonia commonly encountered locally. The two vignettes used featured a father seeking treatment for his 2-year-old son: in the one for diarrhoea, the child had had loose stools for 2 days; in the one for severe pneumonia, the child had had a fever and cough for 5 days and difficulty breathing (full details of the vignettes are available from the corresponding author). Appropriate treatment for diarrhoea was defined according to 2005 World Health Organization guidelines as the provision of oral rehydration solution, with or without zinc supplements and without the prescription of unnecessary or potentially harmful drugs.^26^ The appropriate treatment for severe pneumonia was defined as the provision of antibiotics, without the prescription of potentially harmful drugs, and referral to a hospital for further evaluation.^27^

### Standardized patients

We assessed health-care providers’ performance in practice using a standardized patient method in which enumerators trained as standardized patients visited providers unannounced.^8^^,^^9^^,^^28^^–^^30^ We used a proxy, standardized patient, case method,^8^ in which a father seeks treatment for his 2-year-old son who has the same clinical symptoms described in the vignettes. The cases and methods used for standardized patients were developed by members of our study team and have now been used in numerous settings.^8^^,^^31^^,^^32^ Knowledge and performance scores were calculated for the vignettes and standardized patients, respectively, according to item response theory and based on whether the correct diagnostic questions had been asked and the appropriate examinations had been proposed.^23^^,^^33^

### Statistical analysis

We used a difference-in-difference method to compare changes in providers’ performance between areas in which the programme was implemented and areas in which it was not.^34^^–^^40^ Implementation areas were those in which at least one SkyHealth or SkyCare provider was active, as recorded by our field staff at follow-up data collection. Although the study was originally designed as a large-scale, cluster randomized study,^22^ substantial deviations from the planned randomization occurred. Hence, we employed a quasiexperimental difference-in-difference analysis. Specifically, we used multiple linear regression to explore the association between changes in providers’ knowledge and performance (i.e. outcomes) and an indicator variable denoting the period before or after implementation of the programme interacted with an indicator of whether or not the study cluster was in an implementation area.

The difference-in-difference method provided estimates of covariate-adjusted differences in the knowledge and performance of providers between implementation and nonimplementation areas at follow-up that took into account any differences that existed before implementation. Our estimates probably captured both the direct effects of the programme on participating providers and the indirect effects on other providers in study areas that resulted from market competition. The analysis also included an indicator for the year and district fixed effects to account for unobserved differences across districts at the two assessments. Since, at baseline, it was not possible to predict which providers would participate in the programme, we did not have baseline data for all providers to conduct a provider-level, difference-in-difference analysis. As a robustness check, we performed analysis of covariance (ANCOVA) regressions to compare providers in implementation and nonimplementation areas using follow-up data with district fixed effects and cluster-level average values for outcomes at baseline. In all regressions, standard errors were clustered at the cluster level to correct for the correlation of the error term across providers within the same cluster. We used Stata version 13.1 for all statistical analyses.

Assuming an intracluster correlation of 0.05, a sample of 400 providers (i.e. 5 providers in each of 80 clusters) would yield over 95% power at the 0.05 level of significance for detecting a standardized effect size of 0.4 on knowledge scores based on the vignettes. Similarly, a sample of 200 providers (i.e. 5 in each of 40 clusters) would yield over 99% power for detecting a standardized effect size of 0.66 on performance scores based on the standardized patients. These relatively large effect sizes reflect the projected impact of the programme at the time of planning.

## Results

Overall, 50 of the 80 study clusters were in areas in which the programme was implemented. At baseline and follow-up, providers in implementation and nonimplementation areas were comparable in terms of demographic characteristics, infrastructure, clinical activity and the type of medicine practiced (Table 1; available at: http://www.who.int/bulletin/volumes/95/5/16-179556). However, given the criteria for enrolment in the programme, participating providers were younger than nonparticipating providers, had more experience using computers, had fewer years of clinical experience and reported working fewer hours. Relative to the baseline in 2011, providers in both implementation and nonimplementation areas reported lower patient volumes, longer working hours and higher consultation fees in 2014.

**Table 1:** Health-care providers’ characteristics, social franchising and telemedicine programme, Bihar, India, 2011–2014

Health-care provider’s characteristics	Baseline assessment in 2011 before the programme^a^ was implemented (*n* = 395)			Follow-up assessment in 2014 after the programme^a^ was implemented (*n* = 415)		
	Nonimplementation areas (*n* = 149)	Implementation areas (*n* = 246)		Nonimplementation areas (*n* = 141)	Implementation areas	
					Nonparticipating providers (*n* = 226)	Participating providers (*n* = 48)^b^
Age, mean	45.3	43.3		45.7	42.7	37.8
Proportion educated beyond high school, no. (%)	106 (71.1)	196 (79.7)		114 (80.9)	188 (83.2)	41 (85.4)
Proportion with a medical qualification,^c^ no. (%)	34 (22.8)	47 (19.1)		28 (19.9)	37 (16.4)	7 (14.6)
Proportion who have ever used a computer, no. (%)	32 (21.5)	43 (17.5)		34 (24.1)	50 (22.1)	20 (41.7)
Experience in years, mean	18.7	18.1		18.6	17.0	12.6
Patient caseload per day, mean	19.6	17.5		15.7	14.1	11.3
Working hours per week, mean	49.5	50.2		57.4	57.8	56.0
Proportion who have run camps,^d^ no. (%)	12 (8.1)	19 (7.7)		10 (7.1)	20 (8.8)	9 (18.8)
Proportion working in a public health facility, no. (%)	3 (2.0)	7 (2.8)		1 (0.7)	2 (0.9)	0 (0.0)
Infrastructure index,^e^ mean	0.1	0.1		0.1	0.1	0.1
Consultation fee in Indian rupees,^f^ mean	12.5	11.3		24.3	28.1	23.9
Proportion performing task, no. (%)						
Holding consultations with patients	148 (99.3)	246 (100.0)		141 (100.0)	226 (100.0)	48 (100.0)
Administering treatment	127 (85.2)	213 (86.6)		117 (83.0)	197 (87.2)	43 (89.6)
Selling drugs	78 (52.3)	111 (45.1)		82 (58.2)	148 (65.5)	30 (62.5)
Performing laboratory-related tasks	7 (4.7)	14 (5.7)		3 (2.1)	0 (0.0)	1 (2.1)
Performing administrative tasks	97 (65.1)	147 (59.8)		110 (78.0)	173 (76.5)	35 (72.9)
Owning the health-care business	109 (73.2)	170 (69.1)		134 (95.0)	216 (95.6)	46 (95.8)
Proportion practising type of medicine, no. (%)						
Allopathic medicine	141 (100.0)	226 (100.0)		129 (91.5)	216 (95.6)	47 (97.9)
Homeopathic or Ayurvedic medicine	117 (83.0)	197 (87.2)		35 (24.8)	50 (22.1)	13 (27.1)
Proportion treating disease type, no. (%)						
Diarrhoea	141 (94.6)	238 (96.7)		137 (97.2)	222 (98.2)	44 (91.7)
Pneumonia	126 (84.6)	203 (82.5)		128 (90.8)	206 (91.2)	44 (91.7)

^a^ The World Health Partners’ Sky Program employed social franchising and telemedicine technology to help improve the knowledge and performance of health-care providers.

^b^ Participating providers included 20 SkyHealth providers and 28 SkyCare providers.

^c^ Medical qualifications included MBBS (equivalent to a medical degree in the United States), BAMS, BUMS and BHMS (i.e. bachelor’s degrees in Ayurvedic, Unani and homeopathic medicine, respectively) degrees as well as diplomas in Ayurvedic medicine and some other medical degrees.

^d^ Outreach and public service health-care camps conducted in communities by health-care providers.

^e^ The infrastructure index was derived from the following variables: (i) the availability of electricity; (ii) the availability of back-up power; (iii) the number of consulting rooms; (iv) the number of beds for day observation; (v) the provision of tests; (vi) the ability to perform X-rays; and (vi) possession of a computer system. The index is a normalized sum of the scores for component variables.

^f^ Consultation fees were adjusted to 2001 Indian rupees using the consumer price index.

**Fig. 1:**
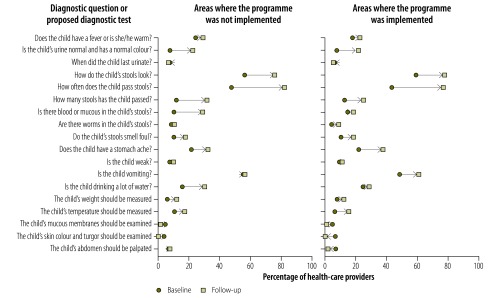
Outcome of a social franchising and telemedicine programme on the questions asked and tests proposed by health-care providers for the childhood diarrhoea vignette, Bihar, India, 2011–2014

Notes: The World Health Partners’ Sky Program employed social franchising and telemedicine technology to help improve the knowledge and performance of health-care providers. Implementation areas included 50 study clusters and nonimplementation areas included 30 study clusters. Health-care providers’ knowledge was assessed at baseline in 2011 before the programme was implemented and at follow-up in 2014 after implementation using a hypothetical patient vignette of childhood diarrhoea as commonly encountered locally, to which they had to respond. Health-care providers in implementation areas were not all participants in the programme.

**Fig. 2:**
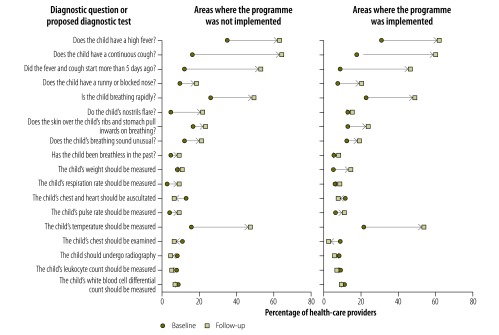
Outcome of a social franchising and telemedicine programme on the questions asked and tests proposed by health-care providers for the childhood pneumonia vignette, Bihar, India, 2011–2014

Notes: The World Health Partners’ Sky Program employed social franchising and telemedicine technology to help improve the knowledge and performance of health-care providers. Implementation areas included 50 study clusters and nonimplementation areas included 30 study clusters. Health-care providers’ knowledge was assessed at baseline in 2011 before the programme was implemented and at follow-up in 2014 after implementation using a hypothetical patient vignette of childhood pneumonia as commonly encountered locally, to which they had to respond. Health-care providers in implementation areas were not all participants in the programme.

**Fig. 3:**
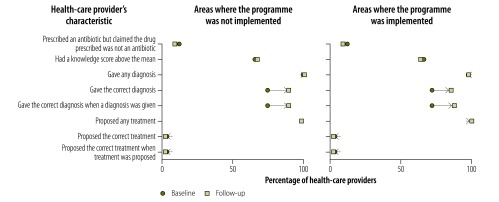
Outcome of a social franchising and telemedicine programme on the diagnosis made and treatment given by health-care providers for the childhood diarrhoea vignette, Bihar, India, 2011–2014

Notes: The World Health Partners’ Sky Program employed social franchising and telemedicine technology to help improve the knowledge and performance of health-care providers. Implementation areas included 50 study clusters and nonimplementation areas included 30 study clusters. Health-care providers’ knowledge was assessed at baseline in 2011 before the programme was implemented and at follow-up in 2014 after implementation using a hypothetical patient vignette of childhood diarrhoea as commonly encountered locally, to which they had to respond. The knowledge score was the item response score based on questions health-care providers asked interviewers in response to the vignette. Health-care providers in implementation areas were not all participants in the programme.

**Fig. 4:**
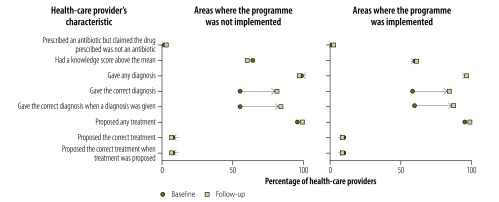
Outcome of a social franchising and telemedicine programme on the diagnosis made and treatment given by health-care providers for the childhood pneumonia vignette, Bihar, India, 2011–2014

Notes: The World Health Partners’ Sky Program employed social franchising and telemedicine technology to help improve the knowledge and performance of health-care providers. Implementation areas included 50 study clusters and nonimplementation areas included 30 study clusters. Health-care providers’ knowledge was assessed at baseline in 2011 before the programme was implemented and at follow-up in 2014 after implementation using a hypothetical patient vignette of childhood pneumonia as commonly encountered locally, to which they had to respond. The knowledge score was the item response score based on questions health-care providers asked interviewers in response to the vignette. Health-care providers in implementation areas were not all participants in the programme.

Providers’ knowledge of childhood diarrhoea and pneumonia was greater at follow-up in both implementation and nonimplementation areas, as illustrated in Fig. 1, Fig. 2 (available at: http://www.who.int/bulletin/volumes/95/5/16-179556), Fig. 3 (available at: http://www.who.int/bulletin/volumes/95/5/16-179556) and Fig. 4: a greater proportion of all types of provider asked diagnostic questions about diarrhoea and pneumonia in response to the vignettes at follow-up than at baseline. However, there was no significant difference between the performance of providers who participated in the programme and those who did not. Fig. 3 and Fig. 4 show data on the diagnoses offered and treatments proposed in response to the diarrhoea and pneumonia vignettes, respectively, at baseline and follow-up. Providers in both implementation and nonimplementation areas gave correct diagnoses more often at follow-up. In implementation areas, the performance of participating providers was generally similar to that of nonparticipants, except that participating providers were more likely: (i) to wrongly report that prescriptions did not include antibiotics; (ii) to prescribe the correct treatment for diarrhoea; and (iii) to diagnose pneumonia in response to the vignette (details available from the corresponding author).

Table 2 shows the results of the difference-in-difference analysis of changes in providers’ knowledge as assessed using the vignettes. The change between baseline and follow-up in the proportion of providers in implementation areas who asked the correct diagnostic questions for pneumonia was significantly less than the change in the corresponding proportion of providers in nonimplementation areas (difference-in-difference estimate: –0.027; 95% confidence interval, CI: –0.054 to –0.001). The programme had no significant effect on any other indicator of providers’ knowledge of either childhood diarrhoea or pneumonia, including making the correct diagnosis, prescribing the correct treatment and prescribing harmful treatments less often. The robustness checks performed using ANCOVA regression analysis showed that the programme had no significant effect on eight of 10 measures of providers’ knowledge (details available from the corresponding author). However, at follow-up, providers in implementation areas were seven percentage points more likely to prescribe the correct treatment in response to the pneumonia vignette than those in nonimplementation areas (intergroup difference: 0.070; 95% CI: 0.015 to 0.129) and seven percentage points less likely to prescribe a harmful treatment (intergroup difference: –0.070; 95% CI: –0.127 to –0.011). These estimates were comparable to point estimates in the difference-in-difference analysis though the latter were not statistically significant.

**Table 2:** Effect of a social franchising and telemedicine programme on health-care providers’ knowledge measured in vignettes, difference-in-difference analysis, Bihar, India, 2011–2014

Knowledge measure	Assessments before and after programme^a^ implementation		Difference-in-difference estimate (95% CI)^b,c^
	Respondents who acted as described		
	Before (*n* = 395)^d^	After (*n* = 405)	
**Diarrhoea vignette^e^**			
Asked the correct diagnostic questions, mean %	21.8	32.1	−0.009 (−0.037 to 0.018)
Made the correct diagnosis, no. (%)	289 (73.2)	356 (87.9)	0.007 (−0.132 to 0.145)
Prescribed oral rehydration solution, no. (%)	287 (72.7)	355 (87.7)	0.063 (−0.050 to 0.176)
Prescribed the correct treatment, no. (%)	15 (3.8)	17 (4.2)	0.021 (−0.034 to 0.075)
Prescribed harmful treatment, no. (%)	355 (89.9)	371 (91.6)	−0.056 (−0.153 to 0.042)
**Pneumonia vignette^e^**			
Asked the correct diagnostic questions, mean %	14.9	34.3	−0.027 (−0.054 to −0.001)
Made the correct diagnosis, no. (%)	225 (57.0)	341 (84.2)	0.012 (−0.126 to 0.151)
Prescribed antibiotics, no. (%)	333 (84.3)	356 (87.9)	−0.075 (−0.196 to 0.046)
Prescribed the correct treatment, no. (%)	33 (8.4)	39 (9.6)	0.055 (−0.017 to 0.128)
Prescribed harmful treatment, no. (%)	340 (86.1)	365 (90.1)	−0.058 (−0.151 to 0.036)

CI: confidence interval.

^a^ The World Health Partners’ Sky Program employed social franchising and telemedicine technology to help improve the knowledge and performance of health-care providers.

^b^ The difference-in-difference method estimated the difference in the change from baseline in 2011 to follow-up in 2014 in providers’ knowledge, as assessed using hypothetical vignette interviews, between implementation areas and nonimplementation areas.

^c^ Each row shows the result of a regression model with the outcome variable specified in the first column. All regressions included the following control variables: (i) age; (ii) education; (iii) medical qualifications; (iv) experience; (v) knowledge score (i.e. the item response score based on questions health-care providers asked interviewers in response to the vignette); (vi) working hours; (vii) patient caseload; (viii) a dummy variable indicating whether the health-care facility organizes camps; (ix) a dummy variable indicating whether the facility is a public health facility; and (x) an index for the clinic’s infrastructure. We also included a binary variable indicating whether the cluster in which the health-care facility was located was in a programme implementation area and we included time and district fixed effects. All standard errors were clustered at the cluster level.

^d^ Data on some analysis variables were missing for 10 assessments.

^e^ Health-care providers’ knowledge was assessed using hypothetical patient vignettes of childhood diarrhoea and pneumonia as commonly encountered locally, to which they had to respond.

**Fig. 5:**
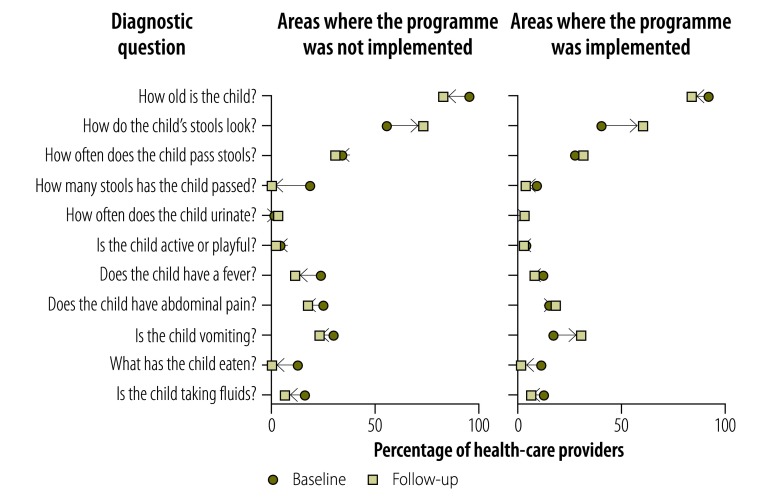
Outcome of a social franchising and telemedicine programme on the questions asked by health-care providers of the childhood diarrhoea standardized patient, Bihar, India, 2011–2014

Notes: The World Health Partners’ Sky Program employed social franchising and telemedicine technology to help improve the knowledge and performance of health-care providers. Implementation areas included 50 study clusters and nonimplementation areas included 30 study clusters. Health-care providers’ performance was assessed at baseline in 2011 before the programme was implemented and at follow-up in 2014 after implementation using a standardized patient method in which enumerators trained as standardized patients visited providers unannounced to ask about their 2-year-old son who was described as having childhood diarrhoea as commonly encountered locally. Health-care providers in implementation areas were not all participants in the programme.

**Fig. 6:**
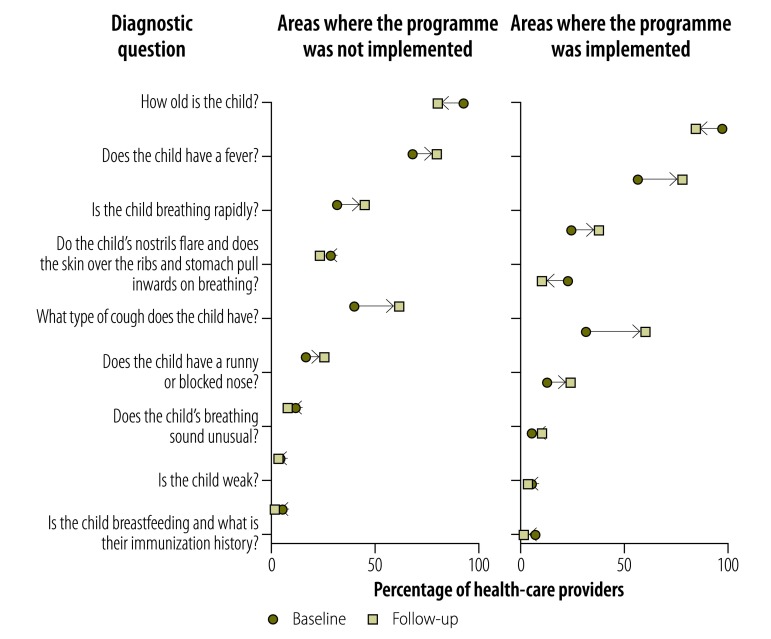
Outcome of a social franchising and telemedicine programme on the questions asked by health-care providers of the childhood pneumonia standardized patient, Bihar, India, 2011–2014

Notes: The World Health Partners’ Sky Program employed social franchising and telemedicine technology to help improve the knowledge and performance of health-care providers. Implementation areas included 50 study clusters and nonimplementation areas included 30 study clusters. Health-care providers’ performance was assessed at baseline in 2011 before the programme was implemented and at follow-up in 2014 after implementation using a standardized patient method in which enumerators trained as standardized patients visited providers unannounced to ask about their 2-year-old son who was described as having childhood pneumonia as commonly encountered locally. Health-care providers in implementation areas were not all participants in the programme.

**Fig. 7:**
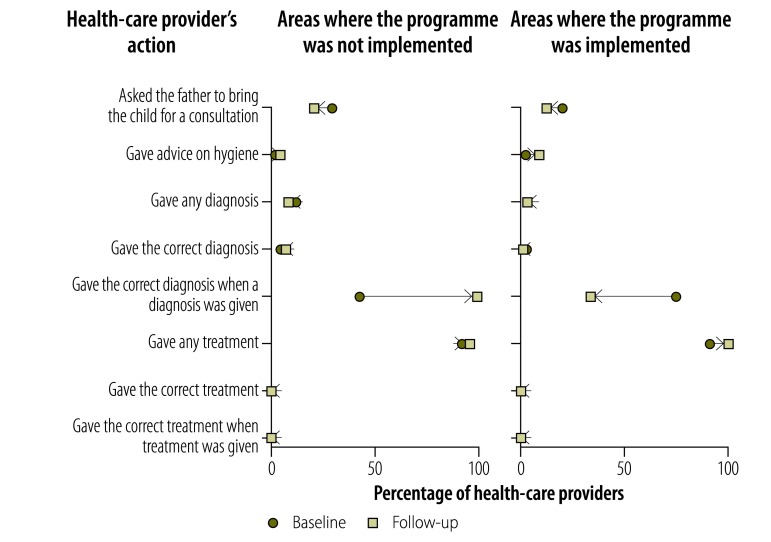
Outcome of a social franchising and telemedicine programme on the diagnosis made and treatment given by health-care providers for the childhood diarrhoea standardized patient, Bihar, India, 2011–2014

Notes: The World Health Partners’ Sky Program employed social franchising and telemedicine technology to help improve the knowledge and performance of health-care providers. Implementation areas included 50 study clusters and nonimplementation areas included 30 study clusters. Health-care providers’ performance was assessed at baseline in 2011 before the programme was implemented and at follow-up in 2014 after implementation using a standardized patient method in which enumerators trained as standardized patients visited providers unannounced to ask about their 2-year-old son who was described as having childhood diarrhoea as commonly encountered locally. Health-care providers in implementation areas were not all participants in the programme.

**Fig. 8:**
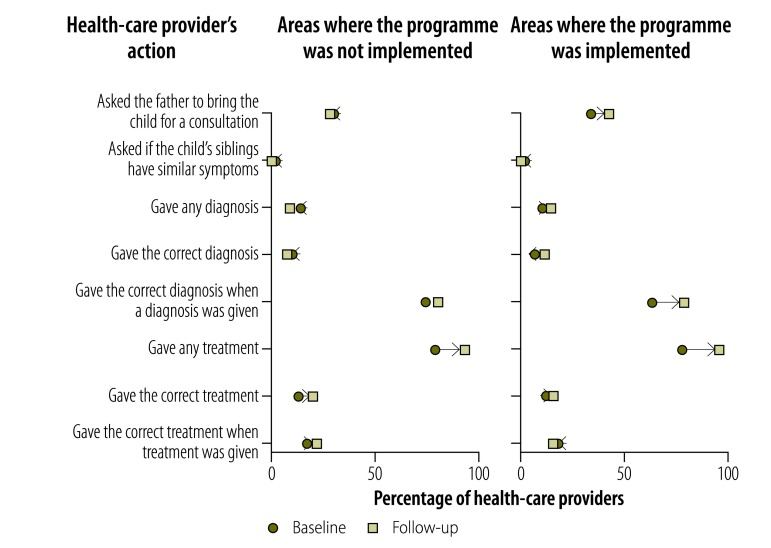
Outcome of a social franchising and telemedicine programme on the diagnosis made and treatment given by health-care providers for the childhood pneumonia standardized patient, Bihar, India, 2011–2014

Notes: The World Health Partners’ Sky Program employed social franchising and telemedicine technology to help improve the knowledge and performance of health-care providers. Implementation areas included 50 study clusters and nonimplementation areas included 30 study clusters. Health-care providers’ performance was assessed at baseline in 2011 before the programme was implemented and at follow-up in 2014 after implementation using a standardized patient method in which enumerators trained as standardized patients visited providers unannounced to ask about their 2-year-old son who was described as having childhood pneumonia as commonly encountered locally. Health-care providers in implementation areas were not all participants in the programme.

Providers spent an average of 1.64 min with the diarrhoea standardized patient at follow-up and prescribed an average of 1.76 drugs; at baseline, the corresponding figures were 1.59 min and 1.85 drugs. Fig. 5 shows that, in general, providers in both implementation and nonimplementation areas asked standardized patients diagnostic questions about diarrhoea less often at follow-up than at baseline, though they asked about the nature of the stools more frequently. For the pneumonia standardized patient, there was no clear pattern in the change between baseline and follow-up in the types of questions asked (Fig. 6; available at: http://www.who.int/bulletin/volumes/95/5/16-179556). Whereas all providers asked more often at follow-up about rapid breathing, the type of cough and a runny nose, providers in programme implementation areas asked less often about chest indrawing with flaring nostrils. The last feature is important because chest indrawing and flaring nostrils are key signs of respiratory distress that can help differentiate severe pneumonia, which is treated with antibiotics and urgent referral to hospital, from pneumonia, which is treated with antibiotics alone. Moreover, these questions are critical given that the child was not present and could not be examined. The performance of providers in giving the correct diagnosis and treatment was poorer with standardized patients than with vignettes, which is consistent with the gap between knowledge and practice previously reported for these providers.^32^ For the diarrhoea standardized patient, all providers prescribed treatment at follow-up but none was the correct treatment for simple diarrhoea (Fig. 7). Among providers in the programme, in contrast, 16.7% (8/48) prescribed the correct treatment at follow-up in response to the diarrhoea vignette. Similarly, 11.1% (1/9) of programme providers prescribed the correct treatment for pneumonia standardized patients at follow up, whereas 22.9% (11/48) proposed the correct treatment in response to the pneumonia vignette. Fig. 8 (available at: http://www.who.int/bulletin/volumes/95/5/16-179556) shows data on the diagnoses offered and treatments prescribed by health-care providers for the pneumonia standardized patient.

Table 3 shows the results of the difference-in-difference analysis of changes in providers’ performance as assessed using standardized patients. There was no evidence for either diarrhoea or pneumonia that the programme was associated with a significant improvement on any measure of provider performance. Robustness checks performed using ANCOVA regression analysis confirmed these findings.

**Table 3:** Effect of a social franchising and telemedicine programme on health-care providers’ performance on standardized patient assessments, difference-in-difference analysis, Bihar, India, 2011–2014

Performance measure	Assessments before and after programme^a^ implementation		Difference-in-difference estimate (95% CI)^b,c^
	Respondents who acted as described^d^		
	Before	After	
**Diarrhoea standardized patient,^e^*n***	178	170	
Asked the correct diagnostic questions, mean %	24.1	22.3	0.052 (−0.014 to 0.118)
Prescribed oral rehydration solution, no. (%)	31 (17.4)	25 (14.5)	−0.104 (−0.243 to 0.035)
Prescribed harmful treatment, no. (%)	159 (89.3)	160 (93.0)	0.002 (−0.147 to 0.152)
Mean performance score	0.0	1.2	0.243 (−0.196 to 0.682)
**Pneumonia standardized patient,**^e^ ***n***	162	163	
Asked the correct diagnostic questions, mean %	30.7	34.3	0.021 (−0.061 to 0.104)
Prescribed antibiotics, no. (%)	104 (64.2)	122 (73.9)	0.067 (−0.183 to 0.318)
Prescribed harmful treatment, no. (%)	106 (65.4)	129 (78.2)	0.137 (−0.087 to 0.362)
Mean performance score	0.0	0.9	0.091 (−0.436 to 0.617)

CI: confidence interval.

^a^ The World Health Partners’ Sky Program employed social franchising and telemedicine technology to help improve the knowledge and performance of health-care providers.

^b^ The difference-in-difference method estimated the difference in the change from baseline in 2011 to follow-up in 2014 in providers’ performance, as assessed in interviews with standardized patients, between implementation areas and nonimplementation areas.

^c^ Each row shows the result of a regression model with the outcome variable specified in the first column. All regressions included the following control variables: (i) age; (ii) education; (iii) medical qualifications; (iv) experience; (v) performance score (i.e. the item response score based on questions health-care providers asked the standardized patient); (vi) working hours; (vii) patient caseload; (viii) a dummy variable indicating whether the health-care facility organizes camps; (ix) a dummy variable indicating whether the facility is a public health facility; and (x) an index for the clinic’s infrastructure. We also included a binary variable indicating whether the cluster in which the health-care facility was located was in a programme implementation area and we included time and district fixed effects. All standard errors were clustered at the cluster level.

^d^ Not all health-care providers were assessed using standardized patients because standardized patients could be introduced without detection only in clinics with a high patient volume.

^e^ Health-care providers’ performance was assessed using a standardized patient method in which enumerators trained as standardized patients visited providers unannounced to ask about their 2-year-old son who was described as having childhood diarrhoea or pneumonia as commonly encountered locally.

## Discussion

We did not find evidence that the World Health Partners’ Sky Program improved the quality of care for childhood diarrhoea or pneumonia in Bihar. Although health-care providers who participated in the programme were almost seven percentage points more likely to propose appropriate treatments with vignettes, we found no significant difference in the quality of care provided in practice for diarrhoea or pneumonia. This failure could be attributed to the weak design and implementation of the programme. For example, only 3 to 6 days of training was offered to providers who signed up as franchisees, which was probably insufficient and was shorter than other training interventions that have successfully improved provider’s knowledge and practice.^13^^,^^41^ For example, a recent study showed that intensive, sustained training for similar informal providers in India significantly improved their performance.^42^ In addition, the programme did not significantly influence patient demand: in 2014, health-care providers in the programme accounted for only 3.5% of all providers in implementation areas and 6% of all private providers. Moreover, fewer than 3% of children with symptoms of diarrhoea or pneumonia were taken to programme providers.^19^

Our study has several limitations. First, assessments of providers were carried out in only 80 randomly selected clusters out of the 360 study clusters. Second, our assessments involved standardized cases of childhood diarrhoea and pneumonia, which means that our findings may not be generalizable to other conditions. However, these two conditions have a high priority in India’s health system.^43^ Another limitation is that, although the standardized patient method is considered the standard for evaluating provider performance,^8^^,^^28^^,^^32^ standardized patients can be introduced without detection only in clinics with a high patient volume (e.g. SkyHealth providers), which excluded most small rural providers who form the majority of SkyCare providers.

Our findings do not necessarily imply that franchising or technology that provides support for clinical decision-making and patient management cannot improve the performance of providers in rural settings. Instead, their failure to improve performance points towards problems with implementation – the programme design may not have been sufficient to boost the quantity or quality of the health-care services delivered. Moreover, because the programme network covered only a small fraction of existing rural health-care providers – most of whom were informally trained, had degrees in alternative medicine or had undertaken apprenticeships with formal practitioners,^44^^,^^45^ it is unlikely that the programme could have influenced health or health care at the population level. Our results also underscore the challenges faced by social franchising in identifying appropriate incentives for providers to adopt technology and improve quality and in understanding patients’ responses to quality improvements.

Social franchising has the potential to create sustainable networks of private health-care providers and improve health-care quality and health outcomes. However, the success of this concept depends on identifying models that can be empirically shown to improve health outcomes before they are scaled up with investments from governments and global donors.

## References

[1] Liu L, Oza S, Hogan D, Perin J, Rudan I, Lawn JE, et al. Global, regional, and national causes of child mortality in 2000-13, with projections to inform post-2015 priorities: an updated systematic analysis. Lancet. 2015 1 31;385(9966):430–40. 10.1016/S0140-6736(14)61698-6 25280870

[2] Lahariya C, Paul VK. Burden, differentials, and causes of child deaths in India. Indian J Pediatr. 2010 11;77(11):1312–21. 10.1007/s12098-010-0185-z 20830536

[3] Chopra M, Mason E, Borrazzo J, Campbell H, Rudan I, Liu L, et al. Ending of preventable deaths from pneumonia and diarrhoea: an achievable goal. Lancet. 2013 4 27;381(9876):1499–506. 10.1016/S0140-6736(13)60319-0 23582721

[4] Bhutta ZA, Das JK, Walker N, Rizvi A, Campbell H, Rudan I, et al.; Lancet Diarrhoea and Pneumonia Interventions Study Group. Interventions to address deaths from childhood pneumonia and diarrhoea equitably: what works and at what cost? Lancet. 2013 4 20;381(9875):1417–29. 10.1016/S0140-6736(13)60648-0 23582723

[5] Chaudhury N, Hammer J, Kremer M, Muralidharan K, Rogers FH. Missing in action: teacher and health worker absence in developing countries. J Econ Perspect. 2006 Winter;20(1):91–116. 10.1257/089533006776526058 17162836

[6] Das J, Hammer J, Leonard K. The quality of medical advice in low-income countries. J Econ Perspect. 2008 Spring;22(2):93–114. 10.1257/jep.22.2.93 19768841

[7] Das J, Hammer J. Money for nothing: the dire straits of medical practice in Delhi, India. J Dev Econ. 2007;83(1):1–36. 10.1016/j.jdeveco.2006.05.004

[8] Das J, Holla A, Das V, Mohanan M, Tabak D, Chan B. In urban and rural India, a standardized patient study showed low levels of provider training and huge quality gaps. Health Aff (Millwood). 2012 12;31(12):2774–84. 10.1377/hlthaff.2011.1356 23213162 PMC3730274

[9] Das J, Kwan A, Daniels B, Satyanarayana S, Subbaraman R, Bergkvist S, et al. Use of standardised patients to assess quality of tuberculosis care: a pilot, cross-sectional study. Lancet Infect Dis. 2015 11;15(11):1305–13. 10.1016/S1473-3099(15)00077-8 26268690 PMC4633317

[10] Grépin K. The role of the private sector in delivering maternal and child health services in low-income and middle-income countries: an observational, longitudinal analysis. Lancet. 2014;384 suppl 1:S7 10.1016/S0140-6736(14)61870-5

[11] Gautham M, Shyamprasad KM, Singh R, Zachariah A, Singh R, Bloom G. Informal rural healthcare providers in North and South India. Health Policy Plan. 2014 7;29 Suppl 1:i20–9. 10.1093/heapol/czt050 25012795 PMC4095923

[12] Das J, Holla A, Mohpal A, Muralidharan K. Quality and accountability in healthcare delivery: audit evidence from primary care providers in India. Am Econ Rev. 2016;106(12):3765–99. 10.1257/aer.20151138 29553219

[13] Das J, Chowdhury A, Hussam R, Banerjee A. The impact of training informal health care providers in India: a randomized controlled trial. Science. 2016;354(6308):aaf7384. 10.1126/science.aaf7384 27846471

[14] Beyeler N, York De La Cruz A, Montagu D. The impact of clinical social franchising on health services in low- and middle-income countries: a systematic review. PLoS One. 2013 4 23;8(4):e60669. 10.1371/journal.pone.0060669 23637757 PMC3634059

[15] Koehlmoos TP, Gazi R, Hossain SS, Rashid M. Social franchising evaluations: a scoping review. London: EPPI-Centre, Social Science Research Unit, Institute of Education, University of London; 2011. Available from: http://eppi.ioe.ac.uk/cms/Default.aspx?tabid=3085 [cited 2017 Mar 15].

[16] Koehlmoos TP, Gazi R, Hossain SS, Zaman K. The effect of social franchising on access to and quality of health services in low- and middle-income countries. Cochrane Database Syst Rev. 2009 1 21; (1):CD007136. 19160323 10.1002/14651858.CD007136.pub2 PMC6791299

[17] Pereira SK, Kumar P, Dutt V, Haldar K, Penn-Kekana L, Santos A, et al. Protocol for the evaluation of a social franchising model to improve maternal health in Uttar Pradesh, India. Implement Sci. 2015 5 26;10(1):77. 10.1186/s13012-015-0269-2 26008202 PMC4448271

[18] Aung T, Montagu D, Su Su Khin H, Win Z, San AK, McFarland W; ORS + Zinc Study Group. Impact of a social franchising program on uptake of oral rehydration solution plus zinc for childhood diarrhea in Myanmar: a community-level randomized controlled trial. J Trop Pediatr. 2014 6;60(3):189–97. 10.1093/tropej/fmt108 24401752

[19] Mohanan M, Babiarz KS, Goldhaber-Fiebert JD, Miller G, Vera-Hernández M. Effect of a large-scale social franchising and telemedicine program on childhood diarrhea and pneumonia outcomes in India. Health Aff (Millwood). 2016 10 1;35(10):1800–9. 10.1377/hlthaff.2016.0481 27702952

[20] The WHP model. Washington DC: World Health Partners; 2014. Available from: https://web.archive.org/web/20140211090501/http:/worldhealthpartners.org/Resources/report_26.pdf [cited 2015 Jul 1].

[21] World Health Partners: leveraging entrepreneurship for health care delivery. Philadelphia: The Wharton School; 2014. Available from: http://knowledge.wharton.upenn.edu/article/world-health-partners-leveraging-entrepreneurship-health-care-delivery/ [cited 2014 Jul 1]

[22] Bihar Evaluation of Social Franchising and Telemedicine (BEST) [Internet]. Bethesda: ClinicalTrials.gov, National Library of Medicine; 2015. Available from: https://clinicaltrials.gov/ct2/show/NCT01345695 [cited 2015 Dec 11].

[23] Das J, Hammer J. Which doctor? Combining vignettes and item response to measure clinical competence. J Dev Econ. 2005;78(2):348–83. 10.1016/j.jdeveco.2004.11.004

[24] Leonard KL, Masatu MC. The use of direct clinician observation and vignettes for health services quality evaluation in developing countries. Soc Sci Med. 2005 11;61(9):1944–51. 10.1016/j.socscimed.2005.03.043 15936863

[25] Peabody JW, Luck J, Glassman P, Jain S, Hansen J, Spell M, et al. Measuring the quality of physician practice by using clinical vignettes: a prospective validation study. Ann Intern Med. 2004 11 16;141(10):771–80. 10.7326/0003-4819-141-10-200411160-00008 15545677

[26] Handbook: IMCI integrated management of childhood illness. Geneva: Department of Child and Adolescent Health and Development, World Health Organization; 2005. Available from: http://apps.who.int/iris/bitstream/10665/42939/1/9241546441.pdf [cited 2017 Mar 15].

[27] Arora N; India Clinical Epidemiology Network(IndiaCLEN) Task Force on Pneumonia. Rational use of antibiotics for pneumonia. Indian Pediatr. 2010 1;47(1):11–8. 10.1007/s13312-010-0015-4 20139472

[28] Rethans J-J, Gorter S, Bokken L, Morrison L. Unannounced standardised patients in real practice: a systematic literature review. Med Educ. 2007 6;41(6):537–49. 10.1111/j.1365-2929.2006.02689.x 17518833

[29] Leonard KL, Masatu MC. Using the Hawthorne effect to examine the gap between a doctor’s best possible practice and actual performance. J Dev Econ. 2010;93(2):226–34. 10.1016/j.jdeveco.2009.11.001

[30] Rethans JJ, Sturmans F, Drop R, van der Vleuten C. Assessment of the performance of general practitioners by the use of standardized (simulated) patients. Br J Gen Pract. 1991 3;41(344):97–9. 2031767 PMC1371620

[31] Sylvia S, Shi Y, Xue H, Tian X, Wang H, Liu Q, et al. Survey using incognito standardized patients shows poor quality care in China’s rural clinics. Health Policy Plan. 2015 4;30(3):322–33. 10.1093/heapol/czu014 24653216

[32] Mohanan M, Vera-Hernández M, Das V, Giardili S, Goldhaber-Fiebert JD, Rabin TL, et al. The know-do gap in quality of health care for childhood diarrhea and pneumonia in rural India. JAMA Pediatr. 2015 4;169(4):349–57. 10.1001/jamapediatrics.2014.3445 25686357 PMC5023324

[33] Leonard KL, Masatu MC, Vialou A. Getting doctors to do their best: the roles of ability and motivation in health care quality. J Hum Resour. 2007;42(3):682–700. 10.3368/jhr.XLII.3.682

[34] Sutton M, Nikolova S, Boaden R, Lester H, McDonald R, Roland M. Reduced mortality with hospital pay for performance in England. N Engl J Med. 2012 11 8;367(19):1821–8. 10.1056/NEJMsa1114951 23134382

[35] Babiarz KS, Miller G, Yi H, Zhang L, Rozelle S. New evidence on the impact of China’s New Rural Cooperative Medical Scheme and its implications for rural primary healthcare: multivariate difference-in-difference analysis. BMJ. 2010 10 21;341:c5617. 10.1136/bmj.c5617 20966008 PMC6173169

[36] Farrar S, Yi D, Sutton M, Chalkley M, Sussex J, Scott A. Has payment by results affected the way that English hospitals provide care? Difference-in-differences analysis. BMJ. 2009 8 27;339:b3047. 10.1136/bmj.b3047 19713233 PMC2733950

[37] Craig P, Cooper C, Gunnell D, Haw S, Lawson K, Macintyre S, et al. Using natural experiments to evaluate population health interventions: new Medical Research Council guidance. J Epidemiol Community Health. 2012 12;66(12):1182–6. 10.1136/jech-2011-200375 22577181 PMC3796763

[38] Basinga P, Gertler PJ, Binagwaho A, Soucat AL, Sturdy J, Vermeersch CM. Effect on maternal and child health services in Rwanda of payment to primary health-care providers for performance: an impact evaluation. Lancet. 2011 4 23;377(9775):1421–8. 10.1016/S0140-6736(11)60177-3 21515164

[39] Wooldridge JM. Econometric analysis of cross section and panel data. 2nd ed. Cambridge: The MIT Press; 2010.

[40] Josua D, Angrist J-SP. Mostly harmless econometrics. 1st ed. Princeton: Princeton University Press; 2009.

[41] Sharma DC. Concern over private sector tilt in India’s new health policy. Lancet. 2015 1 24;385(9965):317. 10.1016/S0140-6736(15)60103-9 25713831

[42] Das J, Chowdhury A, Hussam R, Banerjee AV. The impact of training informal health care providers in India: A randomized controlled trial. Science. 2016 10 7;354(6308):aaf7384. 10.1126/science.aaf7384 27846471

[43] Patel V, Parikh R, Nandraj S, Balasubramaniam P, Narayan K, Paul VK, et al. Assuring health coverage for all in India. Lancet. 2015 12 12;386(10011):2422–35. 10.1016/S0140-6736(15)00955-1 26700532

[44] Holla A. Measuring the quality of health care in clinics. Washington DC: The World Bank Group; 2013. Available from: https://www.globalhealthlearning.org/sites/default/files/page-files/Measuring%20Quality%20of%20Health%20Care_020313.pdf [cited 2017 Mar 15].

[45] Das V. Affliction: health, disease, poverty. New York: Fordham University Press; 2015.

